# Histologic Distribution of Lung and Bronchus Tumors: A Population-Based Study in the Upper Peninsula of Michigan

**DOI:** 10.7759/cureus.55220

**Published:** 2024-02-29

**Authors:** Kyle A Burton, Sheetal Acharya, Matthew T Foley

**Affiliations:** 1 Medicine, Michigan State University College of Human Medicine, East Lansing, USA; 2 Hematology, Upper Peninsula Health Systems, Marquette, USA; 3 Internal Medicine, Michigan State University College of Human Medicine, East Lansing, USA

**Keywords:** bronchus cancer, smoking, lung cancer, michigan, upper peninsula

## Abstract

Introduction: Lung cancer remains the leading cause of cancer-related mortality in the United States, with cigarette smoking recognized as the most important modifiable risk factor. The distinct smoking rates and occupational landscape in the Upper Peninsula of Michigan underscore the necessity of investigating the multifactorial influences on the prevalence and distribution of lung and bronchus cancer within this population.

Methods: This study, conducted from January 2012 to December 2022, included 1035 patients diagnosed with lung or bronchus tumors who were first seen and/or received the first course of treatment at Upper Peninsula Health Systems (UPHS) - Marquette, the largest hospital system in the Upper Peninsula of Michigan and one of only two radiation oncology treatment centers in the Upper Peninsula.

Results: This study demonstrated that the histologic trend of lung and bronchus cancers in a sample of 1035 patients in the Upper Peninsula of Michigan closely resembles that of national averages. Participants with a lifetime history of smoking made up 943 (91.1%) cases of patients diagnosed with lung or bronchus cancers in this study. Lifetime non-smokers only made up 53 (5.1%) cases of patients diagnosed with lung or bronchus cancers. The median age at diagnosis of participants in this study was 70 years.

Conclusion: Our study provides significant insights into the histologic distribution of lung and bronchus cancers within the Upper Peninsula of Michigan, addressing a notable gap in the current literature for this rural and medically underserved population. The histologic distribution of lung and bronchus cancers in this region aligns with national trends. Furthermore, the distinct rates of cigarette smoking in the Upper Peninsula emphasize the critical role of smoking cessation efforts in reducing the burden of lung and bronchus cancers in this region.

## Introduction

Lung and bronchus cancer is the leading cause of cancer deaths in the United States [1]. Cigarette smoking is well-known to be the number one risk factor for the development of lung and bronchus cancers. Multiple other risk factors contribute to the development of lung cancer including exposure to secondhand smoke, advancing age, socioeconomic status, and exposure to radon [2,3].

The Upper Peninsula of Michigan has a significant population of adults who smoke cigarettes. From 2018 to 2021, 21.4% of adults residing in the Upper Peninsula of Michigan reported being current cigarette smokers [4]. In contrast, this population of smokers is almost double the national average of 11.5% of U.S. adults who reported to be current cigarette smokers in 2021 [5].

Rates of cigarette smoking are higher in low socioeconomic status groups [6]. Additionally, low socioeconomic status is linked to failure to quit smoking [7]. Data from the U.S. Census Bureau highlights that between 2014 and 2018, 15.6% of Upper Peninsula residents lived under the poverty line, which may provide a link between the high prevalence of smoking in this population [8].

Current United States Preventive Service Task Force (USPSTF) guidelines at the time of this study recommend annual screening for lung cancer with low-dose computed tomography in adults 50 to 80 years who have a 20-pack-year smoking history and currently smoke or have quit within the past 15 years [9]. In 2021, 8.4% of adults residing in the Upper Peninsula of Michigan met the USPSTF recommendations for lung cancer screening [10].

Furthermore, the Upper Peninsula of Michigan has a substantial mining workforce who predominately extract minerals such as copper, nickel, and iron. Exposure to all three of these minerals has been linked to an increased risk of the development of lung cancer [11-13].

## Materials and methods

Data from this retrospective chart review was obtained through the Upper Peninsula Health System (UPHS) tumor registry located in Marquette, MI. Inclusion criteria were established to encompass patients aged 18 years and older diagnosed with lung or bronchus tumors who were first diagnosed and/or received their first course of treatment at UPHS, Marquette. The dataset was filtered to extract pertinent variables including patient age at diagnosis, histologic subtype of lung or bronchus cancer, smoking habits, type of tobacco use, and history of exposure to tobacco products.

A thorough screening process was employed to ensure data integrity and relevance. Initially, 1236 cases spanning from January 2012 to December 2022 were identified from the registry. Exclusion criteria were applied to eliminate cases with significant missing information, particularly concerning smoking-related variables. Specifically, cases with more than two missing variables pertaining to smoking status, type of tobacco use, or history of tobacco exposure were excluded from the analysis.

Upon application of the exclusion criteria, 201 cases were deemed ineligible due to incomplete registrar information, resulting in their exclusion from the study population. Consequently, a final sample size of 1035 cases was identified and utilized in the subsequent analyses, ensuring a robust and representative dataset for investigating the distribution and correlates of lung and bronchus cancers within the Upper Peninsula population.

## Results

Table 1 reflects the histologic type of lung and bronchus cancer of the 1035 patients included in this study. Non-small cell lung cancer (NSCLC) predominates as the most prevalent histologic type of lung and bronchus cancers in the region, followed by small cell lung cancers. Additional tumors discovered in our study population included neuroendocrine carcinomas, carcinoid tumors, and one case of malignant melanoma present in the lung.

**Table 1:** Histologic type of lung and bronchus tumors tabulated by case numbers and corresponding percentages.

Histologic type of lung and bronchus cancer	Number of cases	Percent
NSCLC	796	76.91%
Small cell lung cancer	204	19.71%
Neuroendocrine carcinoma	29	2.80%
Carcinoid tumor	5	0.48%
Malignant melanoma	1	0.10%

Of the initial 1035 lung and bronchus tumor cases, 796 cases were determined to be NSCLC. These cases were tabulated by histologic subtypes of NSCLC including adenocarcinomas and squamous cell carcinomas among others. Adenocarcinomas predominated as the most prevalent of these cases, followed by squamous cell carcinomas. A total of 93 (12%) cases were NSCLC, not otherwise specified, and were thus unable to be further categorized by histologic subtype. Table 2 reflects the distribution of these histologic subtypes of NSCLC.

**Table 2:** Histologic subtype of NSCLC tabulated by case numbers and corresponding percentages. A total of 796 cases of NSCLC are included in this table. NSCLC, non-small cell lung cancer

Histologic subtypes of NSCLC	Number of cases	Percent
Adenocarcinoma	406	51.01%
Squamous cell carcinoma	277	34.80%
Large cell neuroendocrine carcinoma	5	0.63%
Sarcomatoid carcinoma	4	0.50%
Carcinosarcoma, not otherwise specified	3	0.38%
Adenosquamous carcinoma	8	1.01%
NSCLC, not otherwise specified	93	11.68%

Smoking status and exposure to tobacco products of the study population were investigated. Table 3 reflects the smoking status or exposure to tobacco products in the study cohort. A total of 943 (91.1%) participants diagnosed with lung or bronchus cancer were found to have a history of smoking, whereas only 53 (5.1%) participants had no lifetime history of smoking. Of the 1035 cases, 32 cases had an unknown smoking status. Given the important link between tobacco use and lung cancer, these cases were included to highlight the importance of comprehensive documentation, including detailed tobacco use history, in epidemiologic studies and public health research.

**Table 3:** Smoking status of the 1035 participants included in the study tabulated by number of cases and corresponding percentages.

Smoking status	Number of cases	Percent
Smokers	943	91.11%
Lifetime non-smokers	53	5.12%
Unknown smoking status	32	3.09%
Chewing tobacco	5	0.48%
Secondhand smoke exposure	2	0.19%

**Figure 1:**
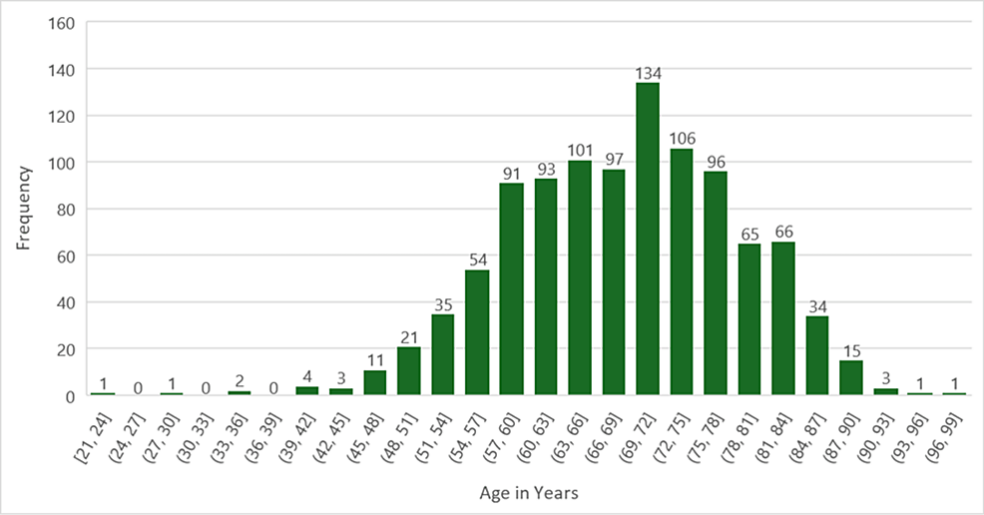
Histogram illustrating the age distribution of the 1035 participants enrolled in this study.

The distribution of age at diagnosis of lung or bronchus cancers in the 1035 participants included in this study is reflected in Figure 1. The most common age range at diagnosis of lung or bronchus cancer in this study population was between 69 and 72 years of age. The median age at diagnosis was found to be 70 years.

**Figure 2:**
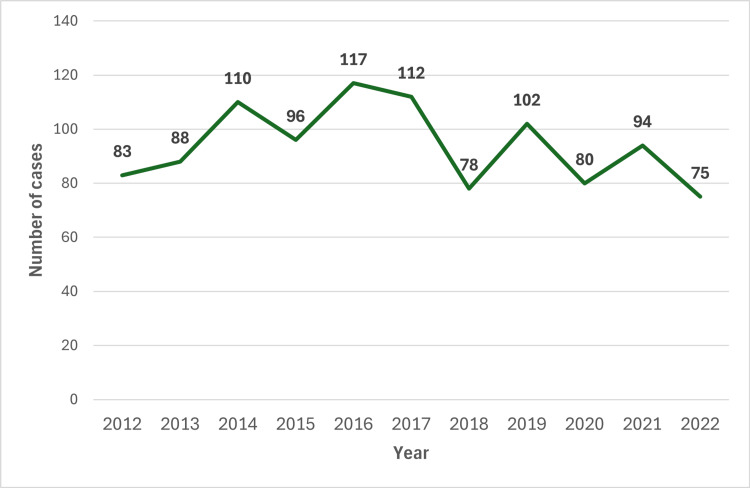
Illustrates the number of lung or bronchus cancer cases diagnosed per year at UPHS, Marquette from January 2012 to December 2022. UPHS, Upper Peninsula Health Systems

Figure 2 reflects the number of lung or bronchus tumor cases diagnosed each year at UPHS, Marquette from January 2012 through December 2022. The average number of lung or bronchus tumor cases diagnosed per year in the study population was 94.1 cases per year.

## Discussion

Our study reveals significant insight into the prevalence of lung and bronchus cancer within the Upper Peninsula of Michigan. This study population comprised patients who were initially diagnosed and/or received their initial course of treatment for lung and bronchus tumors at UPHS, Marquette, the largest hospital system in Michigan's Upper Peninsula and one of just two radiation oncology treatment centers in the area. Marquette, Michigan, the largest city in the Upper Peninsula, had an estimated population of approximately 20786 residents in 2022 [14]. UPHS, Marquette is a hospital facility with a capacity of approximately 222 beds [15]. The substantial size of this hospital system in a sparsely populated region results in a broad catchment area for patients and thus represents an appropriate sample population for this study.

In the United States, about 10%-15% of lung cancers are small cell lung cancers and 80%-85% are NSCLC. Furthermore, approximately 40% of NSCLCs are adenocarcinomas, whereas approximately 25% are squamous cell carcinomas [16]. Histological analysis in our study sample reveals a predominance of NSCLC with adenocarcinoma and squamous cell carcinoma being the most predominant subtypes. Overall, the trends in histologic types of lung and bronchus cancer in the Upper Peninsula align with national averages.

The high prevalence of cigarette smokers in the Upper Peninsula is evidenced by both our data and previous literature. These smoking behaviors correlate with our results that highlight the link between the high prevalence of smokers who subsequently develop lung or bronchus cancers. Additionally, in 2022, 8.4% of adults in Michigan reported using E-cigarettes occasionally or daily, slightly surpassing the national average of 7.7% [17]. While a definitive link between E-cigarette use and lung cancer has not been well established, it is important to continue to monitor the potential health impacts of E-cigarette use [18].

Occupational exposure represents a significant risk factor for lung cancer development [19]. In the Upper Peninsula, a considerable portion of the labor force is engaged in industries such as industrial work, construction, and manufacturing, amplifying the likelihood of occupational exposure in this region [20]. Furthermore, the mining industry, which employs a substantial number of residents, exposes workers to various potential carcinogens, including copper, nickel, and iron. One study indicated that exposure to diesel fumes alone was associated with elevated lung cancer risk [21].

Our results also reveal the number of lung and bronchus cancer cases seen at UPHS, Marquette each year. The trend in the number of cases diagnosed per year has remained relatively stable from January 2012 through December 2022, with an average of 94.1 cases of lung or bronchus cancers diagnosed per year over this period.

The average age at diagnosis among participants in our study was 68.9 years, slightly below the United States' average age at diagnosis of 70 years as supported by current literature [22]. This data signifies the importance of age as a contributing risk factor for the development of cancer, as well as the importance of age-appropriate screening for adult residents in the Upper Peninsula.

Limitations

Limitations of this study include its reliance on the accuracy and completeness of documentation by healthcare professionals and registrars. Cases with incomplete registrar data were excluded, amounting to the exclusion of 201 cases from the analysis. This underscores the critical role of comprehensive documentation in supporting epidemiological studies.

## Conclusions

This study aims to address the gap in lung and bronchus cancer literature originating from the Upper Peninsula of Michigan. Our study finds that the histologic trend of lung and bronchus cancer in the Upper Peninsula of Michigan resembles that of national averages. The predominance of lung and bronchus cancers in this population along with cigarette smoking rates that are notably higher than the national average emphasizes the importance of targeted intervention for smoking cessation and lung cancer screening in this population.
